# Protocol for measuring the responses of multiple budding yeast strains to extracellular change in parallel using a microfluidic device

**DOI:** 10.1016/j.xpro.2025.104071

**Published:** 2025-09-10

**Authors:** Ivan B.N. Clark, Matthew M. Crane, Julian M.J. Pietsch, Peter S. Swain

**Affiliations:** 1 Centre for Engineering Biology, University of Edinburgh, Edinburgh EH9 3BF, Scotland, UK

**Keywords:** Microbiology, Microscopy, Systems biology, Biotechnology and bioengineering

## Abstract

Alcatras microfluidic devices allow long-term, single-cell measurements by immobilizing cells of budding yeast in hundreds of individual traps while removing daughters by the flow of a medium. Here, we present a protocol for simultaneously monitoring the growth and responses of up to five budding yeast strains using a multi-chamber version of the device. We describe steps for preparing logarithmic cultures and medium reservoirs, filling the device, and loading cells. We then detail procedures for securing tubing and starting the medium flow.

For complete details on the use and execution of this protocol, please refer to Granados et al.^1^

## Highlights

• Steps for microfluidic device manufacture (required design file provided)
• Procedures for loading cells from each of the five strains into microfluidic device
• Instructions for delivery and programmed changes of media

## Abstract (editor highlights)

Publisher’s note: Undertaking any experimental protocol requires adherence to local institutional guidelines for laboratory safety and ethics.

## Graphical abstract

**Figure:**
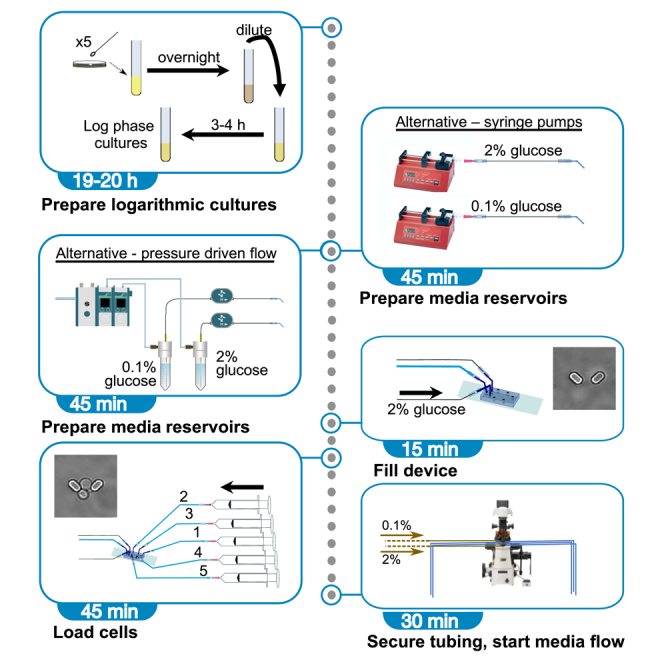

## Before you begin

The following protocol describes the steps needed to observe the nuclear translocation of five *Saccharomyces* transcription factors in response to a rapid drop in the extracellular glucose concentration.^1^ However, the ability to culture 5 strains in parallel, to vary the environment and to observe individual cells over many hours provides many additional applications. We have also used the protocol to investigate the timing of changes in ribosome biogenesis,^2^ to demonstrate real-time control of media conditions in response to changes in cell growth rate^2^ and to study signaling pathways^3^^,^^4^ and metabolic oscillations^5^ in *S.* cerevisiae, as well as to characterize cell cycle arrest and kinetochore protein localization in the human pathogen *Cryptococcus neoformans*.^6^^,^^7^^,^^8^ Variations on the protocol could include different modes of measurement, such as fluorescence intensity rather than localization, ratiometric measures and fluorescence lifetimes. Constant, or gradually changing media conditions could also be imposed rather than a step change.

### Innovation

The microfluidic device used here is a development from a single-chambered design we have described previously,^9^ in which hundreds of cells are immobilized in traps. We have developed a method for introducing cells through the media outlets, and were therefore able to eliminate the cell loading port. Removing this port allows us to fit multiple imaging chambers in a compact space, avoiding the need to move centimeters between fields, while retaining a single-layer design which is relatively cheap and easy to manufacture. Consequently, we can observe up to five independent strains simultaneously in identical conditions. To achieve rapid media switching in a multi-chamber design, we incorporated media bypass channels, based on the “dial-a-wave” junction designed by the Hasty group.^10^ Although the cells are only exposed to one medium at a time, media from the two sources are infused continuously. This prevents any build-up of back pressure which would otherwise result in slower and less predictable media dynamics. The overall design and the protocol are agnostic to the shape of the cell traps. The protocol could therefore be adapted for other cell types, such as diploids or other budding yeast species.

**Figure 1:**
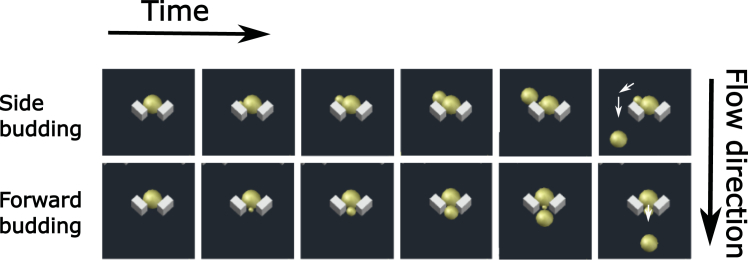
A schematic showing how Alcatras traps release daughter cells Cells lodged between the two pillars of the trap produce buds either with the direction of flow if they bud forwards or against the direction of flow if they bud sideways. In both cases following cytokinesis, the daughters are removed by the flow of medium.

Microfluidic devices are cast in PDMS from a reusable silicon wafer.^11^ The design is based on the Alcatras system,^9^ in which cells are caught between pillars and retained by forces created by the flow of medium. As cells divide asymmetrically, the smaller daughters are removed, either through the gap between the pillars or to the sides, aided by the trapped cell diverting the flow (Figure 1).

**Figure 2:**
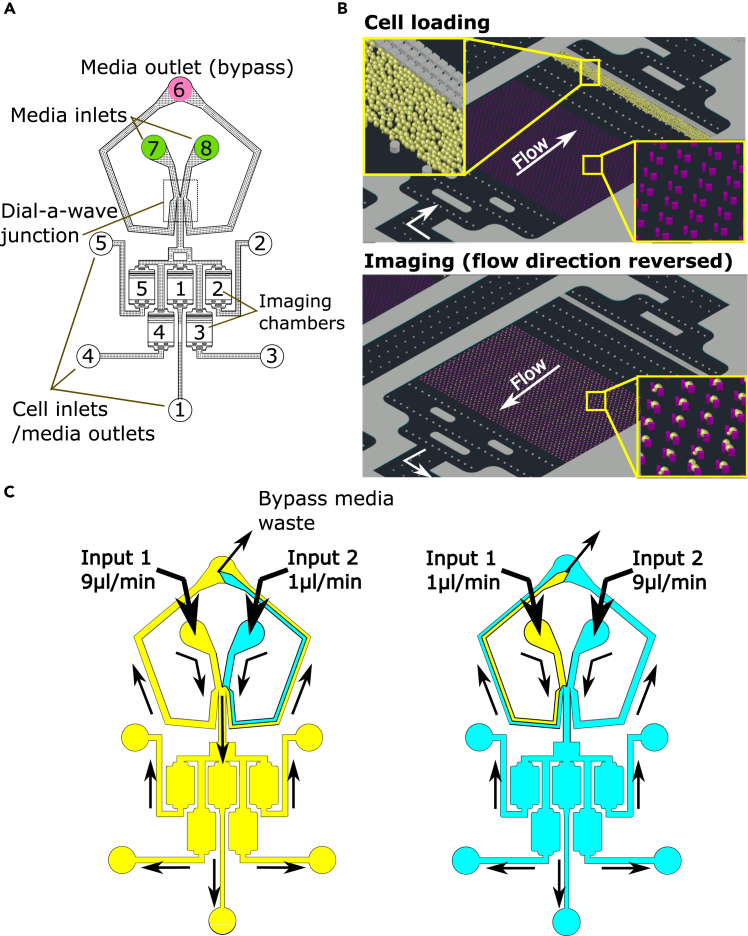
Microfluidic device design and operation (A) Overview. The five imaging chambers are connected to individual ports (white circles, 1–5), which serve as cell inlets during loading and media outlets during operation. There are two media inlet ports (green circles, 7,8), which feed the imaging chambers via a ‘dial-a-wave' junction.^10^ The bypass outlets of the junction feed into a sixth media outlet port (magenta circle, 6). (B) Cell loading and operation. Cell cultures are infused through the cell inlet ports, and cells accumulate at a barrier above the trap array (top inset). The barrier consists of PDMS blocks spaced 2.1 μm apart, which is smaller than the diameter of most cells. During imaging the direction of flow is reversed, and cells are pushed back into the traps (lower inset). (C) Media switching (see also Methods Video S1). Different media are delivered into the device, shown in yellow for the left side (input 1) and cyan for the right (input 2). When input 1 is set to flow at a high rate, the laminar interface between the two media types is driven into the bypass channel to the right, ensuring that all medium flowing to the imaging chambers is from input 1. When the flow rates are reversed, the interface is driven to the left bypass, and cells in the chambers experience the medium from input 2. Arrows show the direction of media flow in different parts of the device.

The design incorporates 5 imaging chambers, into which 5 different strains can be introduced through individual holes (Figure 2A, ports 1–5). The strains are kept separated by barriers at the top of each chamber, where the cells accumulate as they are infused (Figure 2B, upper panel). During operation the direction of flow is reversed and the cells are retained in the chamber traps (Figure 2B, lower panel). Media infused from the two inlets (Figure 2A ports 7 and 8) form a stable, laminar interface. Positioning this interface in one of the bypass channels ensures that cells only experience media from one source at a time. By changing the relative flow rates from each media source, the interface can be moved rapidly (<1s) across the channel leading to the imaging chambers (Figures 2C and Methods Video S1). Media can be delivered by multiple methods; we describe a pressure-based system as well as commonly used syringe pumps.

**Figure 3:**
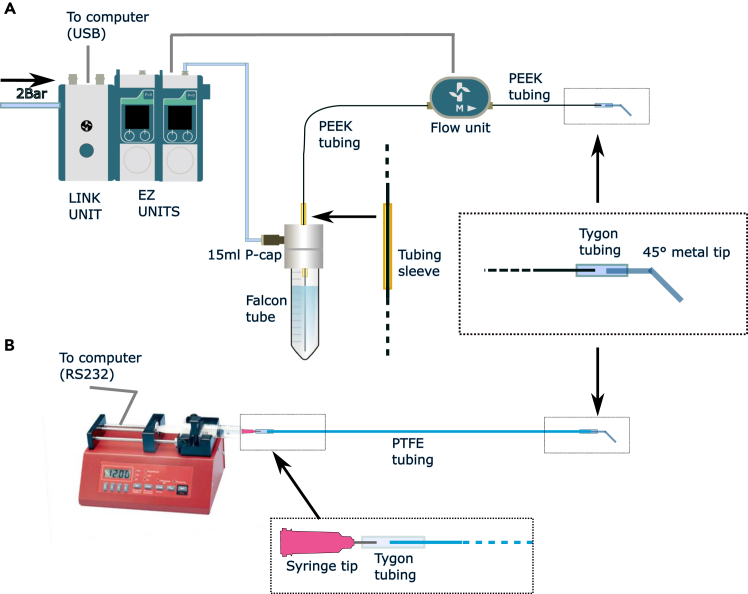
Equipment preparation for imaging and media delivery (A) Overview of tubing and wire connections for media delivery using the Fluigent EZ system. Connections for only one channel are illustrated although two are required. (B) Overview of tubing connections used for media delivery using the Aladdin syringe pump. Connections for only one channel are illustrated although two are required.

Methods Video S1. Media switching principle, related to step 14e (see also Figure 2C)

Methods Video S2. Connecting tubing for delivering cells and media into the microfluidic device, related to steps 7 and 10

### Make microfluidic devices

**Timing: 2 days**

1. Pour PDMS onto wafer mold.
  ***Note:*** Before first use, the wafer must be pre-treated with silane to prevent irreversible adhesion of the PDMS.^12^ It should then be placed in a 140 mm diameter Petri dish with the patterned side facing up, and the steps below followed but with 100 g of PDMS and 10 mL of curing agent. After the PDMS is cut for the first time, edges of the Petri dish will be filled and then 50 g and 5 mL can be used subsequently. The silane treatment should be repeated from time to time when the cured PDMS appears to be adhering more to the wafer at step 2a (approximately after every 10 pourings of PDMS onto the wafer). These steps are summarized in Figure S1.
  a. Mix 50 g of Sylgard 184 (PDMS) with 5 mL curing agent in a disposable cup.
  b. Stir vigorously for 2 to 3 min to ensure uniform curing.
  c. Place the cup in a vacuum desiccator connected to an air pump. Run the pump for 30 min to degas the PDMS.
  d. Pour the degassed PDMS over the wafer in a 140 mm diameter Petri dish.
    ***Note:*** Some air bubbles may form when pouring the PDMS onto the wafer. These will burst when the PDMS is heated at the next step.
  e. Cure the PDMS by baking overnight at 65°C.
2. Cut devices and tube holes.
  a. Cut the PDMS around the edge of the wafer with a scalpel. Carefully peel the PDMS off the wafer surface.
  b. Cut around each device pattern with a scalpel.
  c. Cut a short piece of clear adhesive tape. Reduce the adhesion by pushing a clean gloved thumb onto the tape 3 or 4 times.
    ***Note:*** Pushing a gloved thumb on the tape reduces the adhesion. If the adhesion is too strong the trap pillars may be damaged when the tape is removed.
  d. Stick the tape onto the top (pattered) surface of one PDMS device. The pattern should now be clearly visible.
  e. Using a biopsy punch and cutting mat, cut 1 mm diameter holes vertically through the PDMS at the media and cell inlet and outlet ports (1–8) shown in Figure 2A.
3. Device bonding.
  a. Ensure both of the air valves of the plasma bonding chamber are empty. Close the door and run the vacuum pump for 35 s.
  b. Switch the RF (Radio Frequency) power control from “off” to “high”.
    ***Note:*** Running the plasma protocol with the chamber empty before bonding devices seems to improve bonding efficiency, perhaps because it reduces the water vapor content of the air in the chamber. After switching on the RF, pink fluorescence should be seen through the chamber viewing window
  c. Leave the RF on for 1 min, adjusting the air inlet valve continuously to maximize the brightness of the plasma fluorescence during treatment.
  d. Switch off the RF and the vacuum pump. Open the main air valve to allow the chamber to return to atmospheric pressure so the door can open.
    ***Note:*** When there is a device and cover slip in the chamber, initially the valve must be opened partially so that the influx of air is slow. Otherwise there is a risk that the cover slip will be blown by the air and break.
  e. Remove any debris from the patterned surface of the device by pushing a piece of reduced adhesion tape (see step 2c) onto the surface 3–4 times.
  f. Place the device patterned-surface up in the center of the plasma chamber.
  g. Place a 22 mm x 32 mm cover glass next to the device in the chamber.
  h. Repeat steps 3a to 3d to treat the surfaces for bonding.
  i. Remove the cover slip from the chamber and place flat on the bench with the treated surface up.
  j. Remove the device from the chamber. Turn over so that the treated surface is facing down and place carefully onto the treated cover slip.
  k. Repeat steps 3e to 3j for the remaining devices molded from the wafer.
    ***Note:*** There are several alternative methods for bonding, but the details are beyond the scope of this protocol. The bonded devices can be stored indefinitely before use.

### Prepare strains

**Timing: 2 days**

4. Strain preparation.
  a. From frozen stocks, streak cultures of Msn2-GFP, Dot6-GFP, Maf1-GFP, Mig1-GFP and Sfp1-GFP onto YPD agar plates.
  b. Incubate the plates at 30°C for 2 days to allow colony formation.

### Prepare medium perfusion system

**Timing: 30 min**

***Note:*** Two alternative versions of this step (5 and 6) are presented, for setup using either syringe pumps or the pressure-based Fluigent system. These steps only need to be performed once. The tube connections for each method are summarized (for a single media source in each case) in Figures 3A and 3B. PEEK tubing (outer diameter 1/32 inch) is required for fitting to the Fluigent flow units and can be reused after cleaning (main protocol steps 15–20). Disposable PTFE tubing is used for the syringe pump alternative and the method of tubing preparation is given in the main protocol (step 7).

5. Prepare media perfusion (Fluigent EZ system).
  a. Connect the flow units.
    i. Attach PEEK tubing to the outlet of one of the flow units.
    ii. Cut a 3 cm length of Tygon tubing using scissors that have been sterilized by wiping with 70% ethanol.
    iii. Insert the end of the tubing coming from the flow unit outlet into the Tygon tubing.
    iv. Insert the longer part of a metal tip (see materials and equipment, Methods Video S2 into the Tygon tubing.
    v. To prevent contamination of this tip, secure it in a sterile flask using a foam bung.
      ***Note:*** This end will be inserted into the microfluidic device.
    vi. Attach PEEK tubing to the inlet of the flow unit.
    vii. Repeat steps i–iii for the other flow unit.
  b. Connect the P-caps.
    i. Connect the Fluigent Link unit to two EZ units and to a compressed air source.
    ii. Connect the two flow units to the EZ units using the micro-USB ports.
    iii. Insert the PEEK tubing leading to the inlet of one of the flow units into a tubing sleeve. Connect it to a 15 mL P-cap. Connect the P-cap to one of the EZ flow control units using 4 mm air hose.
    iv. Add 13 mL 70% ethanol to a 15 mL Falcon tube and connect the P-cap, ensuring an air-tight seal around the rubber O-ring.
    v. Push the PEEK tubing down so that it is close to the bottom of the media tube before tightening the fitting at the top.
    vi. Repeat steps i–iii with the other P-cap, flow unit and EZ unit.
    vii. Set the flow rates on both EZ units to 20 μl/min for 10 min to clean and sterilize the tubing and flow units.
6. Prepare media perfusion (syringe pumps).
  a. Connect each syringe pump directly to an RS232 port on your PC via the “to computer” port on the back of the pump.
  b. Connect the power cables of both pumps.
  c. Install and test the pump control script (see materials and equipment below).

## Key resources table

**Table:** 

REAGENT or RESOURCE	SOURCE	IDENTIFIER
**Chemicals, peptides, and recombinant proteins**		

Dow SYLGARD 184 kit	Ellsworth Adhesives	Cat# 0002-01-000032
Trichloro(1H,1H,2H,2H-perfluorooctyl) silane	Sigma-Aldrich	Cat# 448931
Complete supplement mixture	Formedium	Cat# DCS0011
Ammonium sulfate	Merck	Cat# 1.01217.1000
Yeast nitrogen base without amino acids or ammonium sulfate	Fisher Scientific	Cat# 233520
D-glucose	Sigma-Aldrich	Cat # FLUH99C80E9C
Bovine serum albumin	Sigma-Aldrich	Cat# A7030
Cy5 (sulfo-cyanin carboxylic acid, optional)	Abcam	Cat# ab146502

**Experimental models: Organisms/strains**		

Dot6-GFP from yeast GFP clone collection^13^	Edinburgh Genome Foundry	YER088C (BY4741 background)
Maf1GFP from yeast GFP clone collection^13^	Edinburgh Genome Foundry	YDR005C (BY4741 background)
Mig1-GFP from yeast GFP clone collection^13^	Edinburgh Genome Foundry	YGL035C (BY4741 background)
Msn2-GFP from yeast GFP clone collection^13^	Y Edinburgh Genome Foundry	YMR037C (BY4741 background)
Sfp1-GFP from yeast GFP clone collection^13^	Edinburgh Genome Foundry	YLR403W (BY4741 background)

**Other**		

Wafer for casting microfluidic design (height 4.6 μm)	Micro Resist Technology	Custom order (see materials and equipment section). Mask design is available.
Vacuum desiccator chamber	Fisher Scientific	Cat# DES-600-070F
Air pump	KNF	Cat# Laboport N 881
YPD agar plates	Sambrook and Russell^14^	Self-made with materials from Formedium
Stationary incubator for plates	Sanyo	Cat# MIR-262
10 mL syringe	BD	Cat# 305959
0.22-micron syringe filters	Merck	Cat# SLGP033RS
Shaking incubator for liquid cultures	Eppendorf	New Brunswick I 26
Spectrophotometer for optical density measurement	GE Healthcare	80-2116-20R Ultrospec 10
Disposable cup	Many sources	N/A
Scissors or tube cutter	Many sources	N/A
Pliers	Many sources	N/A
Petri dish 144 mm diameter	Nunc	Cat# 249964
Oven for 65°C incubation	Binder	Cat# 9110-0190
Scalpel	Swann-Morton	Cat# 0503
Adhesive tape	Many sources	Scotch “Magic”
Electrical insulation tape	Many sources	N/A
Biopsy punch 1 mm	Kai	Cat# bpp-10f
Cutting mat	Fisher Scientific	Cat # 1527981
Cover glass 22 × 32 mm, thickness 1.5	Menzel Gläser	Cat# MENZBB022032AC13
Plasma cleaner	Harrick Plasma	Cat# PDC-002
LineUp Flow EZ (x2, for pressure infusion alternative)	Fluigent	Cat# LU-FEZ-7000
LineUp LINK module (for pressure infusion alternative)	Fluigent	Cat# LU-LNK-0002
LineUp supply kit (for pressure infusion alternative)	Fluigent	Cat# LU-SPK-0002
Flow unit M package (0–80 μL/min, x2, for pressure infusion alternative)	Fluigent	Cat# FLU-M-DPCK
15 mL pressure cap HP package (x2, for pressure infusion alternative)	Fluigent	Cat# P-CAP15-HP-PCK
PEEK tubing (1/32”” OD x 0.01”” ID, for pressure infusion alternative)	Fisher Scientific	Cat# 15760989
BD Facs clean	Fisher Scientific	Cat# 15875858
Programmable microfluidic syringe pump (“Aladdin”, x2, for syringe pump infusion alternative)	World Precision Instruments	Cat# AL-1002X
PCI serial 2 port adapter (for syringe pump infusion alternative)	StarTech	Cat# PCI2S5502
Ismatec Tygon tubing (ID 0.076 mm)	Fisher Scientific	Cat# 06460-24
PTFE tubing (ID 0.022”/0.56 mm, OD 0.042”/1.07 mm)	VWR	Cat# MFLX06417-21
Blunt dispensing tips with 45° angle, 20G	Fisnar	Cat # 8001159
Wide-field epifluorescence microscope, with incubation system and automated stage positioning	Nikon	Ti-Eclipse

## Materials and equipment

The wafer used for casting the microfluidic device is made by soft lithography using a chrome mask printed with the device design. Mask and wafer manufacture require specialist facilities and are not described here but can be outsourced. A mask file with multiple copies of the device design is available.^15^ The thickness of the photoresist used in the wafer manufacture determines the height of the microfluidic channels, which affects cell retention in the traps and the overlap between adjacent cells, which in turn affects image analysis. For haploid *Saccharomyces cerevisiae* strains from the Yeast GFP Collection used here the height should be 4.6 μm. The height may need to be optimized when working with other strains or species.

A wide-field inverted fluorescence microscope is required with automated control of x, y and z positioning as well as an incubation system to keep the device and input media at 30°C. The microscope must be equipped with light sources and filter sets for GFP, and optionally Cy5, fluorescence imaging. A focus control system (such as the Nikon Perfect Focus System) is useful for time lapse imaging.

We describe two of many possible setups for delivering and switching the media. Cheap syringe pump models may work for experiments in which the media is switched completely between two sources (as here) but are likely to create anomalies such as oscillating flow rates for more complex protocols. We describe here how to use the popular Aladdin microfluidic model, which has a motor with a small step size for greater precision. While syringe pumps are less expensive, a pressure-based system gives more accurate and reproducible results, particularly when generating gradual changes of concentration of media components.

There is a Python script available for driving the switching protocol using Aladdin pumps, through serial communication.^15^ These pumps can be programmed manually but it is convenient to control them using a computer, which must therefore have two available RS232 serial ports. These can be added using a PCI card. When using the pressure-driven FluigentEZ system we use their proprietary OxyGEN software.

We use PTFE or PEEK tubing to deliver media to the microfluidic device. It is convenient to attach a short steel tube to the end of the tubing which can easily be pushed into the hole in the cured PDMS. The 45° angled tubes that come with pink blunt-end dispensing tips (Fisnar) are ideal and can be removed from their plastic syringe connectors using pliers. The tips have an outer diameter of 0.91 mm and can be pushed easily into 1 mm diameter holes cut in the PDMS using a biopsy punch. It is possible also to use gauge 19 or 18 tips which have a wider outer diameter. These are harder to push into the holes, but form a tighter fit as they compress the PDMS. To connect the metal tips to both the PEEK and PTFE tubing we use short lengths of flexible Tygon tubing (Methods Video S3).

Methods Video S3. Preparing media for syringe pump infusion, related to step 7

### Medium recipes

**Table:** Synthetic complete media stock (without carbon source)

Reagent	Final concentration	Amount
Complete supplement mix	1.2×	7 g
Ammonium sulfate	50 mM	25 g
Yeast Nitrogen Base without amino acids or ammonium sulfate	1.2×	8.5 g
Purified water	–	to total volume 4 l

SC stock can be stored for up to 4 weeks at room temperature.

**Table:** Synthetic complete media with 2% glucose

Reagent	Final concentration	Amount
Synthetic complete media stock	1×	18 mL
Glucose stock (20% w/vol)	2% (w/vol)	2 mL
BSA stock (25 mg/mL, only add for perfusion into device, not to pre-growth media)	50 μg/mL	40 μL

**Table:** Synthetic complete media with 0.1% glucose

Reagent	Final concentration	Amount
Synthetic complete media stock	1×	18 mL
Glucose stock (20% w/vol)	0.1% (w/vol)	100 μL
Purified water	–	1.9 mL
BSA stock (25 mg/mL, only add for perfusion into device, not for pre-growth)	50 μg/mL	40 μL
Cy5 stock (20 μg/mL, optional, only for perfusion)	0.1 μg/mL	100 μL

## Step-by-step method details

### Prepare cell cultures

**Timing: 19–20 h**

We start the experiment with cells in logarithmic growth as this growth best approximates the conditions in the microfluidic chambers where medium is constantly replenished. All media contain BSA to reduce adhesion of cells to the PDMS and should be passed through a 0.2 μm filter before use, to ensure sterility and that there are no particles that could block the microfluidic device. Growth times will vary depending on the strains and media conditions.

1. Pick a colony from each of the 5 plates prepared earlier, corresponding to the 5 strains to be included in the experiment, and inoculate into filtered 5 mL SC media + 2% (w/vol) glucose.
2. Incubate overnight (approximately 6 h) at 30°C with shaking.
3. Dilute each of the cultures in 5 mL fresh filtered SC media + 2% (w/vol) glucose to a final optical density of 0.1.

***Note:*** This dilution is intended to bring the culture down to a low density in which there is no competition between cells for resources. We measure optical density using a GE Ultrospec 10. The relationship between cell number and measured OD may vary depending on the equipment used for measurement.

4. Incubate for 3–4 h at 30°C with shaking.

### Prepare medium reservoirs

**Timing: 30–45 min**

5. Prepare media.
  a. Prepare 20 mL of synthetic complete media (SC) containing 2% glucose (w/vol). Add 40 μL of a 25 mg/mL stock of bovine serum albumin (BSA, final concentration 50 μg/mL). Mix thoroughly.
  b. Prepare 20 mL of synthetic complete media (SC) containing 0.1% glucose (w/vol). Add 40 μL of a 25 mg/mL BSA. Mix thoroughly.

***Optional:*** Add 100 μL of cy5 dye (20 μg/mL stock, final concentration 0.1 μg/mL) to the 0.1% media preparation. This dye allows confirmation of the timing of media switching but should not be used in experiments with red fluorescent proteins because of spectral bleed-through.

***Note:*** Two alternative versions of the next step (6 and 7) are presented, for setup using either syringe pumps or the pressure-based Fluigent system.

6. Prepare media reservoirs - Fluigent EZ system.
  a. Filter 13 mL of each media preparation into a 15 mL Falcon tube using a 20 mL capacity syringe and a 0.22 μm syringe filter.
  b. Attach one of the Fluigent 15 mL pressure caps to each of the two Falcon tubes.
  c. Apply a flow rate of 20 μm/min to both flow channels for 5 min to replace any ethanol (used for disinfection, see step 20) in the tubing with media.
7. Prepare media reservoirs – syringe pumps (Methods Videos S2 and S3).
  a. In a sterile environment, remove the plungers from two 10 mL syringes.
  b. Place a sterile narrow-gauge syringe needle or Luer cap on the end of each syringe outlet to block the flow.
  c. Mount the syringe barrels in a rack with the blocked outlet at the bottom.
  d. Filter one of the media preparations into each of the syringes, excluding air bubbles as much as possible.
  e. Insert the plunger a short distance into the top of each syringe. Ensure a seal is formed to prevent media flowing out when the block is removed from the syringe outlet.
  f. Remove the needles or Luer caps from the syringe outlets.
  g. With the outlet held in a waste container, depress the syringe barrels further until they reach the 10 mL mark of each syringe.
  h. Replace the needles or caps on the syringe outlets to keep the media sterile.
  i. Cut two 20 cm lengths of PTFE tubing and four 3 cm lengths of Tygon tubing using sterile scissors.
    ***Note:*** It is better to make angled cuts in the PTFE tubing to generate a sharp point. This makes it easier to push the ends into the Tygon tubing at the next step.
  j. Insert the ends of the PTFE pieces into the small lengths of Tygon tubing forming a tight seal.
  k. Insert a metal tip (see materials and equipment, Methods Video S2) into the Tygon tubing at one end of each of the PTFE tubes.
  l. Insert a complete 20-gauge blunt dispensing tip into the Tygon tubing at the other end of each PTFE tube.
  m. Attach the complete dispensing tips to the syringes prepared in steps 7a–e.
  n. Insert the metal tips at the end of the media tubes into a sterile flask until ready to use.
  o. Apply gentle pressure to the syringes to expel air from the tubing.
  p. Mount the syringes in the syringe pumps.

### Fill the device with media

**Timing: 15 min**

In this step we fill the device with media, excluding air, which could block media flow.

**Figure 4:**
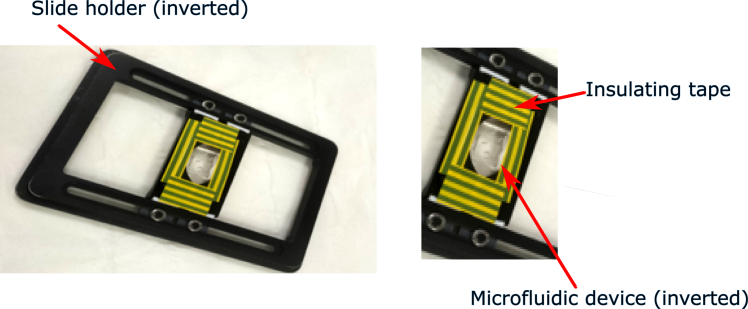
Photographs of the microfluidic device mounted on the underside of a standard microscope specimen holder See also Methods Video S4.

8. Mount a microfluidic device onto the underside of the microscope stage insert.
  a. Secure carefully using electrical insulating tape.
  b. Run a fingernail along the edge of the cover slip to ensure there is no gap between the tape and the surface of the insert (Figure 4, Methods Video S4).

***Note:*** There are multiple possible ways to mount the device on the microscope stage. The following steps require the device to be supported, with the glass of the cover slip resting on a surface. If planning to mount the device on top of a stage insert, then it should not be mounted until after all of the tubes are inserted as it would then be more difficult to support the coverslip when pushing in the metal tips.

9. Fill the microfluidic device with media, excluding air (Methods Video S5).
  a. Insert the metal tips at the ends of the media tubing prepared at stage 6 into the media inlet holes (Figure 2A, holes 7 and 8) of the device.
  b. Cut a 20 cm length of PTFE tubing with sterile scissors making angled cuts.
    ***Note:*** It is better to make angled cuts in the PTFE tubing to generate a sharp point, so that it is easier to push the ends into the Tygon tubing at the next step (see Methods Video S2).
  c. Cut a 3 cm piece of Tygon tubing and insert one end of the PTFE tubing.
  d. Insert a metal tip into the Tygon tubing.
  e. Insert the end of this metal tube into the media bypass waste hole in the device (Figure 2A, hole 6) and put the free end into a flask for waste collection.
  f. Apply a flow rate of 10 μL/min to the media containing 2% glucose using the flow control system (either air pressure-based or syringe pump).
  g. Wait approximately 10 min until small media droplets are visible at all the remaining holes in the device to ensure there is no air in the device.

Methods Video S4. Mounting the microfluidic device on the specimen holder, related to step 8

Methods Video S5. Filling the microfluidic device with media, related to step 9

### Load imaging chambers

**Timing: 45 min**

In this step we load cells from each of the five strains into the device ready for imaging (Methods Videos S6 and S7).

10. Prepare tubing for cell loading.
  a. Switch off the flow in the media inlet tubing.
  b. Cut a 20 cm length of PTFE tubing with sterile scissors, making angled cuts (see note at step 9b).
  c. Attach a 3 cm piece of Tygon tubing to one end.
  d. Insert a metal tip into the Tygon tubing.
  e. Attach another 3 cm piece of Tygon tubing to a 20-gauge blunt syringe tip (without removing the plastic part). Attach to a 10 mL syringe and draw 2–3 mL of air into the syringe.
  f. Insert the free end of the PTFE tubing into the Tygon tubing attached to the syringe.
11. Attach tubing containing cells to each of the cell inlets of the device (Methods Video S6).
  a. Withdraw cell culture to approximately half the length of the PTFE tubing.
    ***Note:*** It can be helpful here to observe the level that the culture has reached by looking for the top of the liquid in the tubing. This helps with monitoring the fluid flow in the following steps.
  b. Release the negative pressure on the plunger. Hold the tip close to the lower hole of the device.
  c. Raise the syringe with your other hand to increase the hydrostatic pressure on the liquid in the tubing. After around 20 s, a small drop of culture should be visible at the end of the steel tip.
  d. If a drop does not appear and the liquid level is not moving down, apply gentle pressure to the syringe plunger until the liquid appears at the end of the tip.
  e. Merge this droplet with the media drop at the lower hole of the device (to exclude air) and insert the steel tip into the hole.
  f. Using a small piece of tissue, absorb any liquid remaining around the hole.
    ***Note:*** Removing the excess liquid here will reduce the probability that these cells contaminate other chambers of the device
  g. Repeat steps 10and 11a–c using the four other cell cultures so there is a tube and syringe connected to each of the 5 cell loading holes.
  h. Label the syringes to distinguish the yeast strains and note which strain is connected to which hole, and therefore which imaging chamber.
12. Load the cells into the device (Methods Video S7).
  a. Mount the device on the microscope stage to observe cell loading.
  b. Focus on the device chambers using a low magnification objective.
  c. Apply gentle pressure to each of the 5 syringes containing cell culture while observing the build-up of cells on the lower surface of the barrier in each chamber (Figure 2B).
    **CRITICAL:** Excessive pressure can drive cells through the barriers at the top of each imaging chamber, resulting in clogging of the device.
    ***Note:*** The pressure may be maintained on each syringe by holding the plunger in position using electrical tape.
  d. Allow cells to accumulate until they reach the first row of supporting columns in the device.
  e. When there are sufficient cells in an imaging chamber, remove the pressure from the syringe that is connected to that chamber.
    **CRITICAL:** Avoid loading too many cells as this can result in device clogging.
  f. Continue cell loading until all of the chambers have enough cells.

Methods Video S6. Connecting cell loading syringes, related to steps 10 and 11

Methods Video S7. Loading the cells, related to step 12

### Complete setup and run image acquisition

**Timing: 6.5 h**

In this step we complete the equipment setup and prepare and start the imaging and media switching protocols.

13. Switch to 60× magnification (Methods Video S8).
  a. Lower the objective, remove the device with attached tubes, then move a high NA 60× objective into position. Apply immersion oil.
  b. Return the device to the microscope stage.
  c. Disconnect the tubing from the five cell loading syringes.
  d. Secure all tubing by taping it to the microscope stage.
    **CRITICAL:** The tubing must be stuck to the stage to avoid any movement or risk of hitting the microscope condenser. However, if the tubing is stretched too tightly it can pull on the metal tips resulting in leaks so the tension on the tubing must be controlled carefully.
    ***Note:*** We secure the tubes in an approximately symmetrical arrangement around the device. This may help to ensure that the sample is flat by balancing any forces from the weight of the tubing.
  e. Insert the ends into conical flasks to collect waste.
14. Run acquisition.
  a. Bring the cells into focus.
  b. Define imaging parameters for capturing z stacks of bright-field, GFP and optionally cy5 images in the microscope acquisition software.
    ***Note:*** The parameters and means of defining these parameters will vary depending on the equipment and software available.
  c. Set the time interval between images to 5 min.
  d. Define stage positions to capture images within each of the 5 device chambers.
  e. Program the delivery of media by the chosen method.
    ***Note:*** The source of media containing 2% glucose should be flowing at 9 μL/min for 3 h, then at 1 μL/min for a further 10 h. The media containing 0.1% glucose should have the reverse pattern, starting at 1 μL/min then switching to 9 μL/min after 3 h. Python code for programming Aladdin syringe pumps is provided for this purpose.^15^
  f. Start the media control program.
  g. Select positions to image and begin image capture.

Methods Video S8. Completing the experiment setup, related to step 13

### Cleaning (pressure-driven flow alternative)

**Timing: 30 min**

Between uses, the PEEK tubing and flow units should be cleaned and sterilized. This could also be performed in the same way with the PTFE tubing used for the syringe pump method though PTFE tubing is cheaper and can also be replaced for each experiment.

15. Fill two 15 mL Falcon tubes with BD Facs clean and two more with 70% ethanol.
16. Replace the media tubes attached to the Fluigent P-caps with the tubes containing Facs clean.
17. Place the metal tips at the ends of the PEEK tubing in a sterile flask to collect waste.
18. Flush each tube (with Facs clean) at 20 μL/min for 10 min.
19. Replace the Falcon tubes attached to the P-caps with the tubes containing 70% ethanol.
20. Flush each tube (with 70% ethanol) at 20 μL/min for 10 min to sterilize.

## Expected outcomes

**Figure 5:**
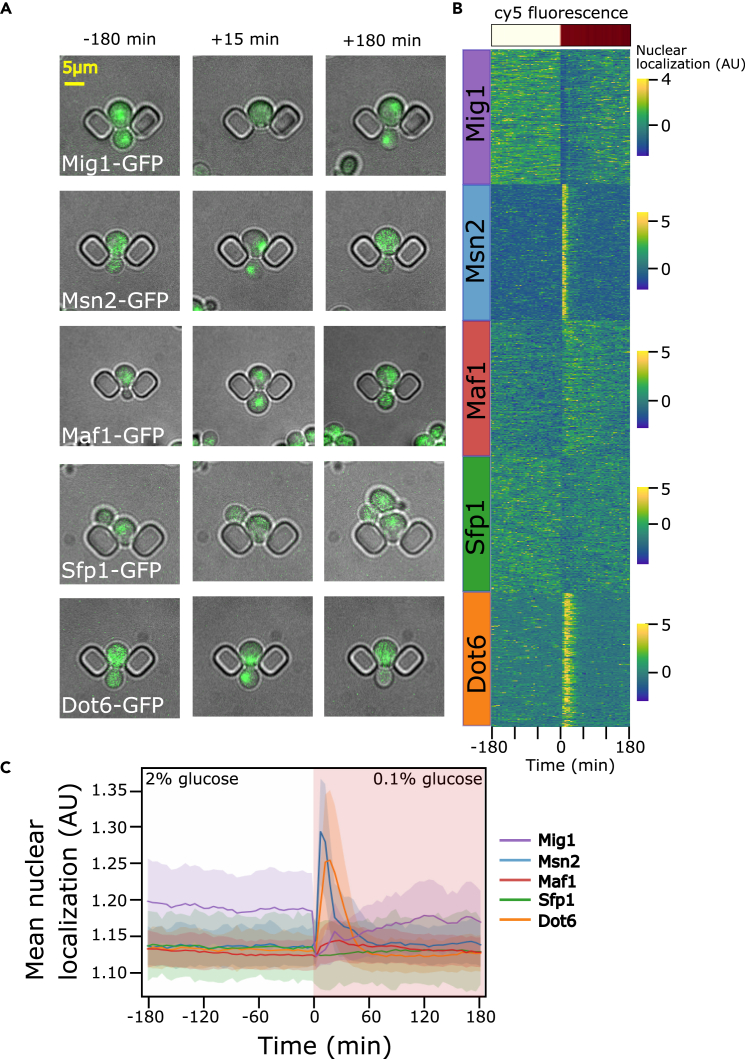
Responses of five transcription factors to a step reduction in extracellular glucose (A) Combined bright-field and fluorescence images showing changes in protein localization for selected cells and time points. The brightness and contrast of the fluorescence channel of each image has been adjusted independently for clarity in showing protein localization. The yellow scale bar represents 5 μm and applies to all of the images. (B) Single cell stress responses. After segmentation and tracking, a pseudorandom sample of 170 cells present throughout the experiment was selected for each strain. The color maps represent the nuclear localization, quantified by the median value of the brightest 5 pixels within each cell area divided by the median of all pixels for the cell.^1^ Individual cells are represented as horizontal lines in the figure, with time as the horizontal axis. The mean cy5 fluorescence of one imaged position is shown with a ‘reds’ colormap above the figure to indicate the time of the media switch. (C) Stress profiles for 5 transcription factors. The median nuclear localization for all cells from each strain is plotted vs time with shading indicating the interquartile range.

This method generates images from which single cell time course data may be extracted. Figure 5 presents data we obtained using the protocol. Because some cells escape from the traps or die during the experiment, we have restricted our analysis to cells that are present and segmented throughout the whole time course. When stress is applied, transcription factors may relocate, either into or out of the nucleus (Figure 5A). A measure of the proportion of nuclear GFP for each cell is shown visually for the five transcription factors (Figure 5C), representing the information encoded by these proteins on the nature and magnitude of the environmental change.^1^ Plotting the medians of these values over all cells of each strain provides a visual time course profile of the population-level response (Figure 5C).

## Quantification and statistical analysis

Cell segmentation and data extraction methods are outside the scope of this protocol, but various algorithms for achieving these goals are publicly available.^2^ To quantify nuclear localization, we create a projection of the maximum values from all GFP focal sections then divide the median fluorescence of the brightest 5 pixels within each cell by the median fluorescence of the cell as a whole. This ratio gives a reliable measure of nuclear accumulation and has been used extensively, although we have recently developed an improved method.^16^

## Limitations

One significant limitation concerns the genetic background of strains that may be cultured in our devices. The trap dimensions have been optimized for the common haploid laboratory strains BY4741 and BY4742,^17^ which are derived from the selected non-flocculent strain S288C. Diploids, and strains of other backgrounds, have different distributions of cell sizes, which will affect the retention of mothers in the traps.^18^ Additionally, diploids bud at both poles which makes them less likely to be retained over multiple divisions. The cells must be flocculation-deficient, and we have also found that the common laboratory strain W303 and its derivatives show high adhesion between mother and daughter cells, preventing their use in our devices. This adhesion may be a consequence of a mutation in *BUD4*, which affects adhesion and colony morphology in the presence of certain other alleles.^19^

The time for which experiments can be run is limited by the volume of the media reservoirs. This volume can be increased by using larger vessels (e.g., 60 mL syringes or Fluigent P-caps designed for 50 mL tubes – Cat #P-CAP-50-HP-PCK).

## Troubleshooting

### Problem 1: Crowding or clogging of the device

In some experiments, cells may become immobilized outside the traps. These cells will divide and eventually form microcolonies, impeding media flow and making cell tracking over time difficult. The microcolonies occur more often in longer experiments, such as studies of ageing, because cells become larger and give rise to larger daughters as they senesce, so they can become constrained between the PDMS ceiling and the coverslip. In shorter experiments this issue can arise if excessive pressure is applied when loading cells, which can increase the height of the device and drive larger cells into the chambers.

### Potential solution

Apply lower pressure when cell loading (step 12). This method can require some patience as it takes longer for cells to load, and we recommend the technique of taping the ends of the loading syringes (rather than holding the pressure manually). Loading fewer cells will also help. To do that you need to remove the loading pressure earlier.

### Problem 2: Cells getting between and/or through the barriers

In some experiments, cells may become inserted between the pillars of the barriers at the top of the chambers, or in the worst cases pass through the barriers. This phenomenon can be recognized as they often accumulate on the top side of the barriers of other chambers. Cells in these positions will divide and eventually block media flow.

### Potential solution

The solution is the same as with problem 1, applying less pressure when loading cells (step 12). Less pressure avoids both stretching the space between the barrier pillars and applying enough force to drive cells between the pillars. If cells are passing through the barriers it likely reflects a problem with device bonding so the bonding procedure should be carefully optimized.

### Problem 3: Debris in the imaging chambers

In some cases, solid material is seen in the imaging chambers. This material can cause problems with cell segmentation and potentially block the media flow.

### Potential solution

There are a few possible causes, with solutions covered in the main text. Debris will be present if the media is not filtered before use (steps 6 and 7), especially when older media is used, as it will contain precipitated crystals of media components that have precipitated. If the PDMS portion of the device is not cleaned carefully (with sticky tape) before bonding (preparation step 3e), then PDMS debris can also be present. Finally, if the BSA concentration is too high then BSA crystals can also be a problem (step 5).

### Problem 4: Leaks

Media leaks can affect experiments because liquid on the top of the device refracts the bright-field light source and threatens the microscope equipment: exposure to salty media can promote erosion or affect electronics. The most common places where leaks occur are the tubing junctions and at the holes where metal tips are inserted into the device.

### Potential solution

We use Tygon tubing to make connections, and after stretching this tubing becomes stiffer with time, which can lead to difficulty making a reliable seal. So, the Tygon tubing should be fresh or at least replaced frequently.

Leaks at the device can be due to irregularities in the holes into which the metal tips are inserted. These irregularities can be due to blunting of the biopsy punches after repeated use, so discarding and replacing punches should alleviate the problem. Leaks due to irregular holes can sometimes be avoided by switching to metal tips with increased diameter (gauge 19 or 18), but in our experience this switch should not be necessary.

Carefully controlling the tube tension at protocol stage 13d is also important to avoid leaks.

### Problem 5: Focus issues

Problems with maintaining focus are common to all time lapse techniques but are more acute here because the tubes attached to the device can apply destabilizing forces to the sample.

### Potential solution

Sample flatness is important to avoid focus problems. Ensure that the specimen holder is clean before mounting the device. Any debris here will prevent the device from being mounted flat. Ensuring the tubes are taped symmetrically around the device may also help. We recommend using a hardware focus maintenance system (such as the Nikon PFS).

## Resource availability

### Lead contact

Further information and requests for resources and reagents should be directed to and will be fulfilled by the lead contact, Ivan B.N. Clark (ivan.clark@ed.ac.uk).

### Technical contact

Technical questions on executing this protocol should be directed to and will be answered by the technical contact, Ivan B.N. Clark (ivan.clark@ed.ac.uk).

### Materials availability

This study did not generate new unique reagents.

### Data and code availability

Source data for Figure 5 is available on request. Similar data has been published previously and is available.^1^

## Acknowledgments

We thank the Edinburgh Genome Foundry for supplying the yeast GFP collection strains and present and former colleagues in the Swain lab for testing the protocol and providing comments on the manuscript. This research was funded in whole, or in part by a Wellcome Trust Bioimaging Technology Development award (grant number 310933/Z/24/Z) and UKRI (grant numbers BB/I00906X/1, BB/M024881/1, BB/W006545/1 and BB/R001359/1). J.M.J.P was also supported by the Leverhulme Trust (RPG-2018-004). For the purpose of open access, the authors have applied a CC BY public copyright licence to any Author Accepted Manuscript version arising from this submission.

## Author contributions

M.M.C., P.S.S., and I.B.N.C., protocol conceptualization. M.M.C. and I.B.N.C., trap design and device design. M.M.C., I.B.N.C., and J.M.J.P., protocol details. J.M.J.P. and P.S.S., experiment conceptualization and design. I.B.N.C., manuscript writing. P.S.S. assisted with manuscript preparation.

## Declaration of interests

The authors declare no competing interests.
