# Supplementary material for: Protocol for measuring the responses of multiple budding yeast strains to extracellular change in parallel using a microfluidic device
Source: STAR Protoc. 2025 Sep 10;6(3):104071. doi: 10.1016/j.xpro.2025.104071 (PMC12491530; doi:10.1016/j.xpro.2025.104071)
Supplement: Document S1. Figure S1 [file mmc1.pdf]

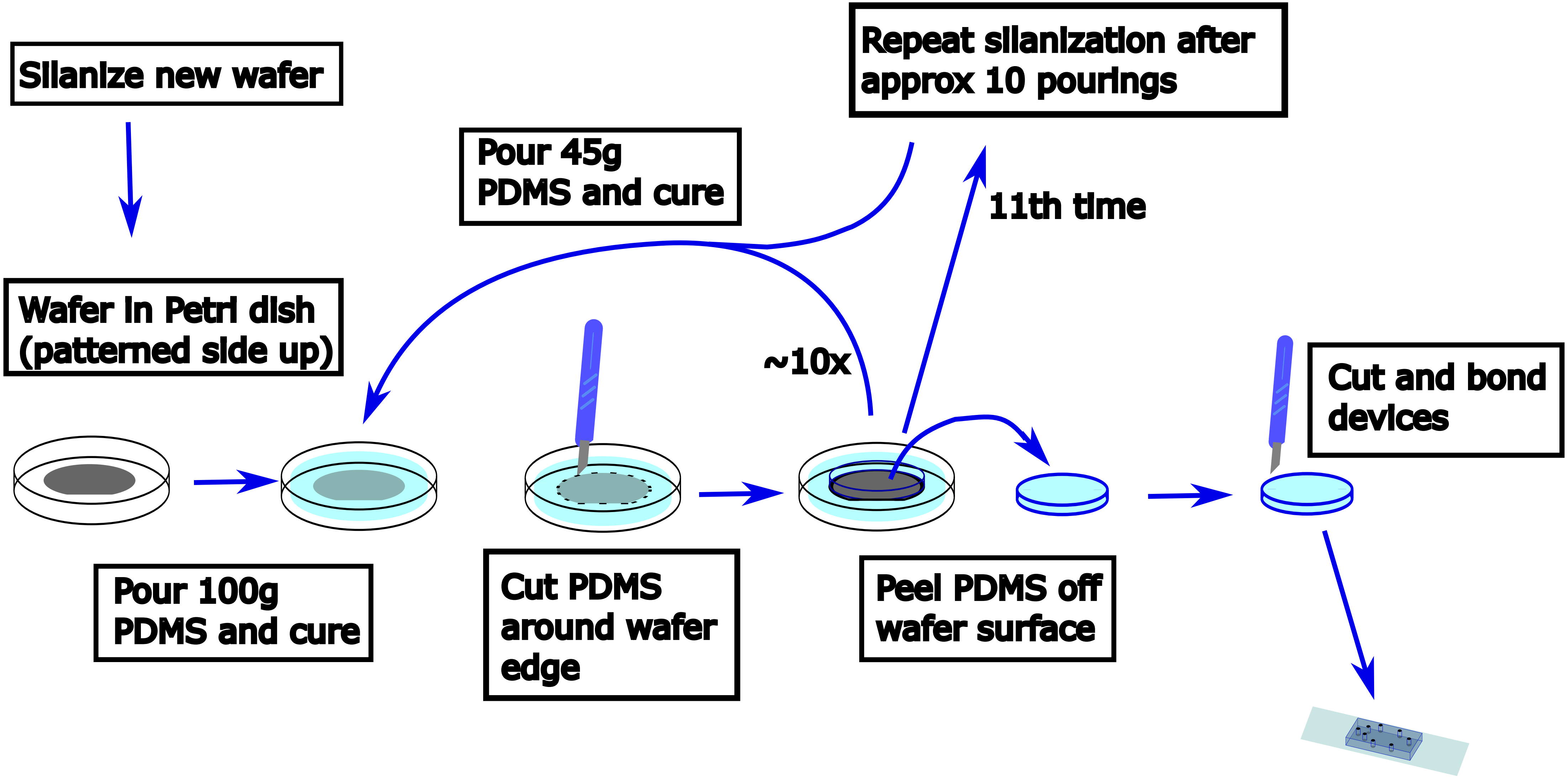

Silanize new wafer
Wafer in Petri dish (patterned side up)
Pour 100g PDMS and cure
Pour 45g PDMS and cure
Cut PDMS around wafer edge
~10x
Repeat silanization after approx 10 pourings
11th time
Peel PDMS off wafer surface
Cut and bond devices

Supplementary figure S1: Device manufacture workflow, related to before you begin, make microfluidic devices. Before first use the wafer must be treated with silane to prevent irreversible PDMS adhesion. We place the silane-treated wafer in a 140 mm diameter Petri dish and pour 100g of de-gassed PDMS (mixed with curing agent), which covers the wafer and also fills the surrounding space in the dish. After removing the PDMS covering the wafer, subsequent pourings require less PDMS (45g) as only the wafer area must be covered. Silane treatment should be repeated approximately after every 10 times the wafer is used
